# How to choose the best journal for your case report

**DOI:** 10.1186/s13256-017-1351-y

**Published:** 2017-07-22

**Authors:** Richard A. Rison, Jennifer Kelly Shepphird, Michael R. Kidd

**Affiliations:** 1 University of Southern California Keck School of Medicine, Los Angeles County Medical Center, 12401 Washington Blvd., Whittier, CA 90602 USA; 2 PIH Health Hospital-Whittier Stroke Center, PIH Health Hospital Non-Invasive Vascular Laboratory, 12401 Washington Blvd., Whittier, CA 90602 USA; 3 JKS Science & Medical Writing, Los Angeles, CA USA; 4 Faculty of Medicine, Nursing and Health Sciences, Flinders University, GPO Box 2100, Adelaide, SA 5001 Australia; 5 Department of Family & Community Medicine, University of Toronto, 500 University Avenue, Toronto, M5G 1V7 Canada

## Abstract

Since the establishment of the *Journal of Medical Case Reports* in 2006, the number of journals that publish case reports has increased rapidly, and most of these journals are open access. Open access publishing usually requires authors to pay publication fees while offering the articles online, free of charge, and free of most copyright and licensing restrictions. The movement for open access has gained support in the research community, with the publishers BioMed Central and PLOS ONE becoming leaders in scientific publishing in their number of articles and citations. As the number of open access publishers has exploded, so too has the number of publishers that act in bad faith to profit from the open access model. Simple guidelines have been developed and resources are available to help authors choose a suitable journal for publication of their case reports.

## Background

Case reports offer unique value to the body of medical knowledge by describing new diseases, disease mechanisms, therapeutic approaches, and adverse or beneficial effects of drugs. The act of recording, discussing with colleagues, and publishing clinical observations as case reports remains essential to the art of medicine and patient care [1]. These short communications generate or enforce hypotheses that may lead to further evaluation in larger study designs [2]. In providing detailed descriptions of the symptoms, signs, diagnosis, treatment, and follow-up of an individual patient, case reports reflect clinical experience and support medical progress. By design, the format lacks statistical sampling, placing it at the bottom of the hierarchy of clinical evidence. Case reports do not include controls, have limited sample size (one to a few individuals), and are unblinded, limitations that require a cautious approach to interpretation of findings. General medical journals publish case reports sparingly, often only publishing those that provide new information on adverse events that can be linked to an intervention [3, 4]. Journal editors may limit inclusion of case reports because they are cited less often than meta-analyses and randomized controlled trials, which negatively affects a journal’s impact factor.

The merits of large randomized studies are well known, but many clinicians recognize the value of case reports as a complement to evidence-based medicine. The case-based nature of clinical practice often is at odds with the population-based nature of research studies, where the findings may have little relevance to an individual patient. Narrow inclusion criteria and the absence of co-morbidities in randomized trials often create a disconnection between typical patient populations and populations represented in research studies [3]. Case reports provide enough detail on one or a small number of patients for clinicians to relate to their own practice. They are educational and interesting to read. For the challenging and patient-centered task of reporting on individual cases with inherent heterogeneous human variability in clinical research and the goal of applicability to real-life circumstances, the CARE guidelines provide a framework for completeness and transparency in case reports. The guidelines aid in finding the balance between adequate detail and concise writing [5].

In response to renewed interest and acknowledgment of their value, the number of peer-reviewed journals that publish case reports has increased in recent years to more than 160 [6]. In the digital era of paperless journals with few space restrictions, the case report has seen a resurgence. The digital format facilitates searches, which is a key factor in their utility [7]. Most of the case report journals are open access and have high acceptance rates. As the number of new scientific journals increases, so do the number of questionable publishers that mislead researchers regarding fees, peer review, and academic credentials. The process of submitting scientific work for publication now includes the need for thorough vetting of potential publishers.

## New case report journals

**Fig. 1:**
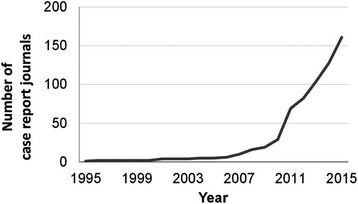
Number of case report journals by year. The number of journals that publish case reports has increased rapidly since 2007. (Reprinted with permission from Akers [6])

In line with the growing demand for case report publishing opportunities, the number of new peer-reviewed journals that focus on case reports had increased to more than 160 journals produced by 78 publishers by mid-2015. Figure 1 shows that the number of case report journals increased rapidly beginning in 2007, a timeframe that coincides with the Great Recession of the late 2000s and the concomitant decline in federal and other funding for basic science and other research. Some of the new journals cover general medicine and others cover specific therapeutic areas. Most case report journals (94%) are open access and approximately 40% are indexed in PubMed. Clinical issues covered by case report journals include previously unreported adverse effects of drugs or other treatments, unexpected events that occur in the course of observing or treating a patient, observations on disease pathogenesis, presentations and/or management of new and emerging diseases, new therapeutic approaches, ethical challenges in patient management, and strategies for preventing or overcoming medical errors [6, 8].

Open access publishing offers freely available and unrestricted use of research and scholarship, which many researchers see as vital to efficient dissemination of science in the digital world [9]. The open access model usually requires authors to pay submission and publication fees upon acceptance, typically between US $300 and $1200 [6]. The move toward making scholarly publications more accessible through open access has continued to gain supporters among the research community. The open access publisher BioMed Central launched in 2000 with 231 articles published that year in 60 journals. In 2015, the numbers increased to more than 30,000 articles in over 290 journals. In 2014, BioMed Central articles were accessed more than 277 million times and had 426,000 citations [10]. Similarly, the number of publications from the open access publisher PLOS ONE, increased from 138 at its inception in 2006 to 28,107 in 2015 [11].

**Table 1:** Case report journals

Journal title	Publisher/Society	Year launched	Open access	PubMed indexed
*A&A Case Reports*	Wolters Kluwer Health/International Anesthesia Research Society	2013	No	No
*AACE Clinical Case Reports*	American Association of Clinical Endocrinologists	2015	Yes	No
*ACG Case Reports Journal*	American College of Gastroenterology	2013	Yes	Yes
*AJP Reports*	Thieme Medical Publishers	2011	Yes	No
*American Journal of Cancer Case Reports*	Ivy Union Publishing	2013	Yes	No
*American Journal of Case Reports*	International Scientific Information	2001	Yes	Yes
*Aperito Journal of Case Reports: Clinical*	Aperito Online Publishing	2015	Yes	No
*APSP Journal of Case Reports*	EL-MED-Pub Publishers/Association of Paediatric Surgeons of Pakistan	2010	Yes	Yes
*Austin Cardio & Cardiovascular Case Reports*	Austin Publishing Group	2015	Yes	No
*Austin Gynecology Case Reports*	Austin Publishing Group	2015	Yes	No
*Austin Journal of Clinical Case Reports*	Austin Publishing Group	2014	Yes	No
*Austin Oncology Case Reports*	Austin Publishing Group	2015	Yes	No
*Autopsy and Case Reports*	Hospital Universitario of the University of San Paulo	2011	Yes	No
*BJR Case Reports*	British Institute of Radiology	2015	Yes	No
*BMJ Case Reports*	BMJ Publishing Group	2008	No	
*Case Reports in Anesthesiology*	Hindawi Publishing	2011	Yes	Yes
*Case Reports in Cardiology*	Hindawi Publishing	2011	Yes	Yes
*Case Reports in Clinical Medicine*	Scientific Research Publishing	2012	Yes	No
*Case Reports in Critical Care*	Hindawi Publishing	2011	Yes	Yes
*Case Reports in Dermatological Medicine*	Hindawi Publishing	2011	Yes	Yes
*Case Reports in Dermatology*	Karger	2009	Yes	Yes
*Case Reports in Emergency Medicine*	Hindawi Publishing	2011	Yes	Yes
*Case Reports in Endocrinology*	Hindawi Publishing	2011	Yes	Yes
*Case Reports in Gastroenterology*	Karger	2007	Yes	Yes
*Case Reports in Gastrointestinal Medicine*	Hindawi Publishing	2011	Yes	Yes
*Case Reports in Genetics*	Hindawi Publishing	2011	Yes	Yes
*Case Reports in Hematology*	Hindawi Publishing	2011	Yes	Yes
*Case Reports in Hepatology*	Hindawi Publishing	2011	Yes	Yes
*Case Reports in Immunology*	Hindawi Publishing	2011	Yes	Yes
*Case Reports in Infectious Diseases*	Hindawi Publishing	2011	Yes	Yes
*Case Reports in Internal Medicine*	Sciedu Press	2014	Yes	No
*Case Reports in Medicine*	Hindawi Publishing	2009	Yes	Yes
*Case Reports in Nephrology*	Hindawi Publishing	2011	Yes	Yes
*Case Reports in Nephrology and Dialysis*	Karger	2011	Yes	Yes
*Case Reports in Neurological Medicine*	Hindawi Publishing	2011	Yes	Yes
*Case Reports in Neurology*	Karger	2009	Yes	Yes
*Case Reports in Obstetrics and Gynecology*	Hindawi Publishing	2011	Yes	Yes
*Case Reports in Oncological Medicine*	Hindawi Publishing	2011	Yes	Yes
*Case Reports in Oncology*	Karger	2008	Yes	Yes
*Case Reports in Ophthalmological Medicine*	Hindawi Publishing	2011	Yes	Yes
*Case Reports in Ophthalmology*	Karger	2010	Yes	Yes
*Case Reports in Orthopedics*	Hindawi Publishing	2011	Yes	Yes
*Case Reports in Otolaryngology*	Hindawi Publishing	2011	Yes	Yes
*Case Reports in Pancreatic Cancer*	Mary Ann Liebert Inc. Publishing	2015	Yes	No
*Case Reports in Pathology*	Hindawi Publishing	2011	Yes	Yes
*Case Reports in Pediatrics*	Hindawi Publishing	2011	Yes	Yes
*Case Reports in Perinatal Medicine*	De Gruyter	2012	Optional	No
*Case Reports in Plastic Surgery and Hand Surgery*	Taylor & Francis/Acta Chirurgica Scandinavica Society	2014	Yes	Yes
*Case Reports in Psychiatry*	Hindawi Publishing	2011	Yes	Yes
*Case Reports in Pulmonology*	Hindawi Publishing	2011	Yes	Yes
*Case Reports in Radiology*	Hindawi Publishing	2011	Yes	Yes
*Case Reports in Rheumatology*	Hindawi Publishing	2011	Yes	Yes
*Case Reports in Surgery*	Hindawi Publishing	2011	Yes	Yes
*Case Reports in Transplantation*	Hindawi Publishing	2011	Yes	Yes
*Case Reports in Urology*	Hindawi Publishing	2011	Yes	Yes
*Case Reports in Vascular Medicine*	Hindawi Publishing	2011	Yes	Yes
*Case Reports in Women’s Health*	Elsevier	2014	Yes	No
*Case Reports International*	Edorium Journals	2012	Yes	No
*Case Reports: Open Access*	Jscholar	2015	Yes	No
*Case Study and Case Report*	Sageya Publishing	2011	Yes	No
*CEN Case Reports*	Springer/Japanese Society of Nephrology	2012	Optional	No
*Clinical Case Reports*	Wiley	2013	Yes	Yes
*Clinical Case Reports and Reviews*	Open Access Text	2015	Yes	No
*Clinical Cases in Mineral and Bone Metabolism*	CIC Edizioni Internazionali/Italian Society of Orthopaedics and Medicine	2004	Yes	Yes
*Clinical Medicine Insights: Case Reports*	Libertas Academia	2008	Yes	Yes
*Clinics and Practice*	PAGEPress	2011	Yes	Yes
*Cold Spring Harbor Molecular Case Studies*	Cold Spring Harbor Laboratory Press	2015	Yes	No
*Dermatology Case Reports*	OMICS International	2015	Yes	No
*Diabetes Case Reports*	OMICS International	2015	Yes	No
*Endocrinology, Diabetes, & Metabolism Case Reports*	Bioscientifica	2013	Yes	Yes
*Epilepsy & Behavior Case Reports*	Elsevier	2013	Yes	Yes
*European Journal of Case Reports in Internal Medicine*	European Federation of Internal Medicine	2014	Yes	No
*European Journal of Pediatric Surgery Reports*	Thieme Medical Publishers	2013	Yes	Yes
*European Journal of Surgical Cases*	Bilimsel Tip Yayinevi	2010	Yes	No
*Experimental and Clinical Endocrinology & Diabetes Reports*	Thieme Medical Publishers	2014	Yes	No
*Global Journal of Medical and Clinical Case Reports*	PeerTechz	2014	Yes	No
*Grand Rounds*	e-MED	2001	Yes	No
*Gynecologic Oncology Reports*	Elsevier	2011	Yes	Yes
*HeartRhythm Case Reports*	Elsevier/Heart Rhythm Society	2015	Yes	No
*Human Pathology: Case Reports*	Elsevier	2014	yes	No
*IDCases*	Elsevier	2014	Yes	No
*IJSS Case Reports & Reviews*	IJSS Group of Journals/Society of Malaysian Medical Association’s Medical Students and European Medical Student’s Association	2014	Yes	No
*Indian Journal of Medical Case Reports*	CIBTech	2012	Yes	No
*Interdisciplinary Neurosurgery: Advanced Techniques and Case Management*	Elsevier	2014	Yes	No
*International Journal of Advances in Case Reports*	McMed International	2014	Yes	No
*International Journal of Case Reports and Images*	Edorium Journals	2010	Yes	No
*International Journal of Case Reports in Medicine*	IBIMA Publishing	2012	Yes	No
*International Journal of Case Studies*	unclear	2012	Yes	No
*International Journal of Clinical Case Studies*	Graphy Publications	2014	Yes	No
*International Journal of Clinical Cases and Investigations*	unclear	2010	Yes	No
*International Journal of Medical and Pharmaceutical Case Reports*	ScienceDomain International	2014	Yes	No
*International Journal of Surgery Case Reports*	Elsevier	2010	Yes	Yes
*International Medical Case Reports Journal*	Dove Medical Press	2008	Yes	Yes
*JAAD Case Reports*	Elsevier/American Academy of Dermatology	2015	Yes	No
*Jacobs Journal of Clinical Case Reports*	Jacobs Publishers	2015	Yes	No
*JBJS Case Connector*	STRIATUS Orthopaedic Communications	2011	No	No
*JCRS Online Case Reports*	Elsevier/American Society of Cataract and Refractive Surgery and European Society of Cataract and Refractive Surgeons	2013	Yes	No
*JMM Case Reports*	Microbiology Society	2014	Yes	No
*Joseph Journal of Clinical Studies and Medical Case Reports*	Joseph Publishing Group	2015	Yes	No
*Journal of Anaesthesia & Critical Care Case Reports*	International Academic Research Group	2015	Yes	No
*Journal of Cardiology Cases*	Elsevier/Japanese College of Cardiology	2010	No	No
*Journal of Case Reports*	unclear	2011	Yes	No
*Journal of Case Reports and Clinical Research Studies*	VRJ Publishers	2014	Yes	No
*Journal of Case Reports and Images in Medicine*	Edorium	2015	Yes	No
*Journal of Case Reports and Images in Obstetrics and Gynecology*	Edorium	2015	Yes	No
*Journal of Case Reports and Images in Oncology*	Edorium	2015	Yes	No
*Journal of Case Reports and Images in Pathology*	Edorium	2015	Yes	No
*Journal of Case Reports and Images in Surgery*	Edorium	2015	Yes	No
*Journal of Case Reports and Studies*	Annex Publishers	2013	Yes	No
*Journal of Case Reports in Medicine*	Ashdin Publishing	2012	Yes	No
*Journal of Case Reports in Oncology and Therapy*	EJourPub	2015	Yes	No
*Journal of Case Reports in Practice*	Saman Publishing	2013	Yes	No
*Journal of Clinical & Medical Case Reports*	Avens Publishing Group	2013	Yes	No
*Journal of Clinical and Translational Endocrinology Case Reports*	Elsevier	2015	Yes	No
*Journal of Clinical Case Reports*	OMICS International	2011	Yes	No
*Journal of Clinical Studies & Medical Case Reports*	Herald Scholarly Open Access	2014	Yes	No
*Journal of Dermatological Case Reports*	Specjaliści Dermatolodzy	2007	No	No
*Journal of Investigative Medicine High Impact Case Reports*	SAGE Publications	2013	Yes	No
*Journal of Knee Surgery Reports*	Thieme Medical Publishers	2013	Yes	No
*Journal of Medical Case Reports*	BioMed Central	2007	Yes	Yes
*Journal of Medical Cases*	Elmer Press	2010	Yes	No
*Journal of Neurological Surgery Reports*	Thieme Medical Publishers	2012	Yes	Yes
*Journal of Orthopaedic Case Reports*	Indian Orthopaedic Research Group	2011	Yes	No
*Journal of Pediatric Surgery Case Reports*	Elsevier	2013	Yes	No
*Journal of Radiology Case Reports*	EduRad Publishing	2008	Yes	Yes
*Journal of Surgical Case Reports*	Oxford University Press	2010	Yes	Yes
*Journal of Surgical Technique and Case Report*	Wolters Kluwer Health	2014	Yes	Yes
*Journal of Vascular Surgery Cases*	Elsevier/Society for Vascular Surgery	2015	Yes	No
*JPRAS Open*	Elsevier/British Association of Plastic Reconstructive and Aesthetic Surgeons	2015	Yes	No
*JSM Clinical Case Reports*	JSciMed Central	2013	Yes	No
*Medical Case Studies*	Academic Journals	2010	Yes	No
*Medical Mycology Case Reports*	Elsevier/International Society for Human and Animal Mycology	2012	Yes	Yes
*MOJ Clinical & Medical Case Reports*	MedCrave	2015	Yes	No
*Neurocase*	Taylor & Francis	1995	Optional	Yes
*NMC Case Report Journal*	Japan Neurosurgical Society	2014	Yes	No
*OA Case Reports*	OA Publishing London	2012	yes	No
*Oncology & Cancer Case Reports*	OMICS International	2015	Yes	No
*Open Journal of Clinical and Medical Case Reports*	unclear	2015	Yes	No
*Oral and Maxillofacial Surgery Cases*	Elsevier	2015	yes	No
*Oxford Medical Case Reports*	Oxford University Press	2014	Yes	Yes
*Pathology Case Reviews*	Wolters Kluwer Health	1996	No	No
*Pediatric Urology Case Reports*	Hayrettin Ozturk	2014	Yes	No
*Radiology Case Reports*	Elsevier/University of Washington	2006	Yes	No
*Respiratory Medicine Case Reports*	Elsevier	2008	Yes	Yes
*Respirology Case Reports*	Wiley/Asian Pacific Society of Respirology	2013	Yes	Yes
*Retinal Cases and Brief Reports*	Wolters Kluwer Health	2007	Optional	Yes
*SAGE Open Medical Case Reports*	SAGE Publications	2013	Yes	Yes
*Scholarena Journal of Case Reports*	Scholarena	2014	Yes	No
*Scholars Journal of Medical Case Reports*	SAS Publishers	2013	Yes	No
*Southeast Asian Journal of Case Report and Review*	Sageya Publishing	2012	Yes	No
*Surgical Case Reports*	Springer/Japan Surgical Society	2015	Yes	No
*The Thoracic and Cardiovascular Surgeon Reports*	Thieme Medical Publishers	2014	Yes	Yes
*Translational Medicine Case Reports*	Elsevier/European Society for Translational Medicine	2015	Yes	No
*Trauma Case Reports*	Elsevier	2015	Yes	No
*Urology Case Reports*	Elsevier	2013	yes	No
*World Journal of Clinical Cases*	Baishideng Publishing Group	2013	Yes	Yes
*World Journal of Medical and Surgical Case Reports*	Narain Publishers	2012	Yes	No

Reprinted with permission from Akers [6]

## Controversial journals and publishers

As scientific publishing shifts from a business model of subscription revenue to open access, the number of open access journals has exploded. However, the proliferation of journals that will publish seemingly anything for a fee has caused alarm among many in the global research community. Alongside many respected open access publishers, others have entered the space acting in bad faith. Some see it as the “dark side” of open access, a growing collection of pseudo-academic, prestigiously titled journals, many of which have similar but not quite identical websites and names to those of well-known established journals. Many of the websites look sufficiently impressive that non-experts doing online research have trouble distinguishing credible research from junk. Experienced academics have been misled into submitting manuscripts and even serving on editorial boards for pseudo-academic journals, agreements that often are difficult to undo. Most of these journals do not post their publication fees, and often authors are not informed of fees until after submitting a manuscript. Withdrawal of a manuscript, which is necessary before submitting the same paper to a legitimate journal, may require payment of the high fees first [12]. For some authors, this means their work may be lost essentially to the disreputable publisher. Many researchers have complained about poorly executed or absent peer review, hidden fees for submission and publication, and unapproved inclusion of researchers’ names on editorial boards.

Jeffrey Beall, a librarian and associate professor at Auraria Library at the University of Colorado, Denver, coined the term “predatory open access publishing” to describe this situation. He is a critic of open access publishing, blaming the system for creating the problem of predatory publishers. His blog *Scholarly Open Access,* although removed by Beall for unknown reasons in January 2017, closely monitored the increasing number of open access publishers and alerted readers to individuals, publishers, publications, meetings, and scholarly metrics that, in the view of Mr Beall, appeared to exploit the open access model [13]. He maintained a list of “potential, possible, or probable predatory scholarly open-access publishers” and another list of standalone journals. His criteria for inclusion on the lists were derived from the *Code of Conduct for Journal Publishers* from the Committee on Publication Ethics (COPE), and *Principles of Transparency and Best Practice in Scholarly Publishing* from COPE, the Open Access Scholarly Publishers Association (OASPA), and the World Association of Medical Editors [14–16]. Similarly, information in these communications may help authors to discern whether they can trust a particular publisher or journal. The Federal Trade Commission (FTC) in the USA has taken notice of questionable publication practices. In August 2016 it filed a suit against the OMICS Group, a global conglomerate based in India that publishes more than 700 open access journals. The suit claimed that the OMICS Group misled researchers, particularly with regard to their peer-review process (or lack thereof) and high fees that were not readily apparent to authors upon manuscript submission [17]. The purpose of the lawsuit, according to the FTC, is to better inform authors of publishing fees and to have a more transparent peer-review system [18]. The case is still to be litigated in federal court in Nevada at the time of writing this article.

The challenge for watchdogs and authors alike is to decide when a publisher is untrustworthy or simply unprofessional. Some publishers may fall under suspicion due to poor copy editing or amateurish website design, but this may not reflect an outright neglect of scholarly standards. It is important not to blacklist startup publishers who lack experience. Another problem with maintaining lists of disreputable publishers is that because copycat journals are often short-lived, the blacklist will continue to grow but individual entries may quickly become obsolete.

## Choose the right journal: Think. Check. Submit.

The “Think. Check. Submit.” campaign arose in response to concerns about publishing practices, and the effort is supported by a coalition of scholarly publishing organizations. “Think. Check. Submit.” takes a positive approach to help researchers identify credible journals, providing up-to-date guidance for choosing where to publish [18, 19]. To ascertain whether a journal is trusted, authors are advised to follow this checklist:

- Do you or your colleagues know the journal?
  - Have you read any articles in the journal before?
  - Is it easy to discover the latest papers in the journal?
- Can you easily identify and contact the publisher?
  - Is the publisher name clearly displayed on the journal website?
  - Can you contact the publisher by telephone, email, and post?
- Is the journal clear about the type of peer review it uses?
  - Does the journal site explain what these fees are for and when they will be charged?
- Do you recognize the editorial board?
  - Have you heard of the editorial board members?
  - Do members of the editorial board mention the journal on their own websites?
- Is the publisher a member of a recognized industry initiative?
  - Do they belong to the COPE?
  - If the journal is open access, is it listed in the Directory of Open Access Journals (DOAJ)?
  - If the journal is open access, does the publisher belong to the OASPA?
  - Is the publisher a member of another trade association?

In addition to consulting colleagues and academic librarians for journal suggestions, authors have available to them several online resources. BioMed Central previously collaborated with Edanz, a company that assists authors in navigating the publication process, to create the author academy [10]. The free online guide describes best practices in writing and publishing a manuscript, including sections on choosing a journal, writing the manuscript, and publication ethics, among others. BioMed Central now contracts with Nature Research Editing Services and American Journal Experts, both of which offer similar services [20, 21].

Several automated search tools help identify suitable journals as well. Authors insert keywords from their manuscript abstract into a search engine, which then compares the words to many online publications and Edanz Journal Selector covers a broad range of journals. The online tool is free, and Edanz also offers a journal selection service (US $300) in which experts use their publication experience to identify up to four of the best journals for a given paper [22]. The Journal/Author Name Estimator (Jane) focuses on biomedical science journals by searching the Medline database published by the US National Library of Medicine [23]. Other online services offered by publishers Springer and Elsevier suggest journals from their own extensive catalogues [24, 25].

## Impact factor

Journal impact factors, calculated and published by Thomson Reuters, measure the average number of citations per published article for papers published over a 2-year period. Despite the fact that the simple metric can be misleading, the impact factor has become, over time, a marker of journal prestige and desirability. The judgment of a paper’s value is often based more on the journal in which it appears than on its content. Many researchers contend that reliance on impact factors undervalues disciplines or study designs, such as case reports, which have lower citation rates. Overall, the number of citations of an article is commensurate with hierarchies of evidence, with meta-analyses receiving more citations than any other study design. Case reports typically receive few citations, although there are notable exceptions [26]. The number of citations of an article, however, does not necessarily reflect how widely the article has been read or the dissemination of the findings in mainstream media [27].

Efforts to embrace a broader view of value in scientific communication, and perhaps diminish the influence of impact factors, have emerged. Journals of the American Society for Microbiology (ASM) no longer advertise impact factors on their websites. Similarly, in recognizing that impact factors are just one of a number of metrics, Nature journals list a suite of citation-based metrics. Only one case report journal, Taylor & Francis’s *Neurocase*, has received an impact factor (1.124), dating back to 1998.

Medicine/National Institutes of Health Indexed research databases are often curated to ensure the quality of included publications. Clarivate Analytics (formerly Thomson Reuters) offers The Web of Science™, as one such example, and recently introduced the “Emerging Sources Citation Index” to complement their more selective indexes. This collection reflects the growing number of peer-reviewed publications of regional importance and in emerging fields [28].

In conclusion, the growth in number of case report journals has provided authors multiple avenues for publication but, at the same time, it has introduced a new level of uncertainty in the journal selection process. Factors to consider when choosing a journal are: the topics the journal covers, the target audience, length restrictions, and the time to publication. Open access publications, such as the *Journal of Medical Case Reports* from BioMed Central, offer high visibility, relatively rapid publication, and transparent publication policies. The reputation of the journal plays an increasingly important part of the decision, requiring thorough vetting of potential journals.
