# Erratum to: How to choose the best journal for your case report

**DOI:** 10.1186/s13256-017-1452-7

**Published:** 2017-10-05

**Authors:** Richard A. Rison, Jennifer Kelly Shepphird, Michael R. Kidd

**Affiliations:** 1 University of Southern California Keck School of Medicine, Los Angeles County Medical Center, 12401 Washington Blvd, Whittier, CA 90602 USA; 2 PIH Health Hospital-Whittier Stroke Center, PIH Health Hospital Non-Invasive Vascular Laboratory, 12401 Washington Blvd, Whittier, CA 90602 USA; 3 JKS Science & Medical Writing, Los Angeles, CA USA; 4 Faculty of Medicine, Nursing and Health Sciences, Flinders University, GPO Box 2100, Adelaide, SA 5001 Australia; 5 Department of Family & Community Medicine, University of Toronto, 500 University Avenue, Toronto, M5G 1V7 Canada

## Erratum

In the publication of this article [1], there are two errors in Table 1 Case report journals.

**Table 1:** Case report journals

Journal title	Publisher/Society	Year launched	Open access	PubMed indexed
*A&A Case Reports*	Wolters Kluwer Health/International Anesthesia Research Society	2013	No	No
*AACE Clinical Case Reports*	American Association of Clinical Endocrinologists	2015	Yes	No
*ACG Case Reports Journal*	American College of Gastroenterology	2013	Yes	Yes
*AJP Reports*	Thieme Medical Publishers	2011	Yes	No
*American Journal of Cancer Case Reports*	Ivy Union Publishing	2013	Yes	No
*American Journal of Case Reports*	International Scientific Information	2001	Yes	Yes
*Aperito Journal of Case Reports: Clinical*	Aperito Online Publishing	2015	Yes	No
*APSP Journal of Case Reports*	EL-MED-Pub Publishers/Association of Paediatric Surgeons of Pakistan	2010	Yes	Yes
*Austin Cardio & Cardiovascular Case Reports*	Austin Publishing Group	2015	Yes	No
*Austin Gynecology Case Reports*	Austin Publishing Group	2015	Yes	No
*Austin Journal of Clinical Case Reports*	Austin Publishing Group	2014	Yes	No
*Austin Oncology Case Reports*	Austin Publishing Group	2015	Yes	No
*Autopsy and Case Reports*	Hospital Universitario of the University of San Paulo	2011	Yes	No
*BJR Case Reports*	British Institute of Radiology	2015	Yes	No
*BMJ Case Reports*	BMJ Publishing Group	2008	Optional	Yes
*Case Reports in Anesthesiology*	Hindawi Publishing	2011	Yes	Yes
*Case Reports in Cardiology*	Hindawi Publishing	2011	Yes	Yes
*Case Reports in Clinical Medicine*	Scientific Research Publishing	2012	Yes	No
*Case Reports in Critical Care*	Hindawi Publishing	2011	Yes	Yes
*Case Reports in Dermatological Medicine*	Hindawi Publishing	2011	Yes	Yes
*Case Reports in Dermatology*	Karger	2009	Yes	Yes
*Case Reports in Emergency Medicine*	Hindawi Publishing	2011	Yes	Yes
*Case Reports in Endocrinology*	Hindawi Publishing	2011	Yes	Yes
*Case Reports in Gastroenterology*	Karger	2007	Yes	Yes
*Case Reports in Gastrointestinal Medicine*	Hindawi Publishing	2011	Yes	Yes
*Case Reports in Genetics*	Hindawi Publishing	2011	Yes	Yes
*Case Reports in Hematology*	Hindawi Publishing	2011	Yes	Yes
*Case Reports in Hepatology*	Hindawi Publishing	2011	Yes	Yes
*Case Reports in Immunology*	Hindawi Publishing	2011	Yes	Yes
*Case Reports in Infectious Diseases*	Hindawi Publishing	2011	Yes	Yes
*Case Reports in Internal Medicine*	Sciedu Press	2014	Yes	No
*Case Reports in Medicine*	Hindawi Publishing	2009	Yes	Yes
*Case Reports in Nephrology*	Hindawi Publishing	2011	Yes	Yes
*Case Reports in Nephrology and Dialysis*	Karger	2011	Yes	Yes
*Case Reports in Neurological Medicine*	Hindawi Publishing	2011	Yes	Yes
*Case Reports in Neurology*	Karger	2009	Yes	Yes
*Case Reports in Obstetrics and Gynecology*	Hindawi Publishing	2011	Yes	Yes
*Case Reports in Oncological Medicine*	Hindawi Publishing	2011	Yes	Yes
*Case Reports in Oncology*	Karger	2008	Yes	Yes
*Case Reports in Ophthalmological Medicine*	Hindawi Publishing	2011	Yes	Yes
*Case Reports in Ophthalmology*	Karger	2010	Yes	Yes
*Case Reports in Orthopedics*	Hindawi Publishing	2011	Yes	Yes
*Case Reports in Otolaryngology*	Hindawi Publishing	2011	Yes	Yes
*Case Reports in Pancreatic Cancer*	Mary Ann Liebert Inc. Publishing	2015	Yes	No
*Case Reports in Pathology*	Hindawi Publishing	2011	Yes	Yes
*Case Reports in Pediatrics*	Hindawi Publishing	2011	Yes	Yes
*Case Reports in Perinatal Medicine*	De Gruyter	2012	Optional	No
*Case Reports in Plastic Surgery and Hand Surgery*	Taylor & Francis/Acta Chirurgica Scandinavica Society	2014	Yes	Yes
*Case Reports in Psychiatry*	Hindawi Publishing	2011	Yes	Yes
*Case Reports in Pulmonology*	Hindawi Publishing	2011	Yes	Yes
*Case Reports in Radiology*	Hindawi Publishing	2011	Yes	Yes
*Case Reports in Rheumatology*	Hindawi Publishing	2011	Yes	Yes
*Case Reports in Surgery*	Hindawi Publishing	2011	Yes	Yes
*Case Reports in Transplantation*	Hindawi Publishing	2011	Yes	Yes
*Case Reports in Urology*	Hindawi Publishing	2011	Yes	Yes
*Case Reports in Vascular Medicine*	Hindawi Publishing	2011	Yes	Yes
*Case Reports in Women’s Health*	Elsevier	2014	Yes	No
*Case Reports International*	Edorium Journals	2012	Yes	No
*Case Reports: Open Access*	Jscholar	2015	Yes	No
*Case Study and Case Report*	Sageya Publishing	2011	Yes	No
*CEN Case Reports*	Springer/Japanese Society of Nephrology	2012	Optional	No
*Clinical Case Reports*	Wiley	2013	Yes	Yes
*Clinical Case Reports and Reviews*	Open Access Text	2015	Yes	No
*Clinical Cases in Mineral and Bone Metabolism*	CIC Edizioni Internazionali/Italian Society of Orthopaedics and Medicine	2004	Yes	Yes
*Clinical Medicine Insights: Case Reports*	Libertas Academia	2008	Yes	Yes
*Clinics and Practice*	PAGEPress	2011	Yes	Yes
*Cold Spring Harbor Molecular Case Studies*	Cold Spring Harbor Laboratory Press	2015	Yes	No
*Dermatology Case Reports*	OMICS International	2015	Yes	No
*Diabetes Case Reports*	OMICS International	2015	Yes	No
*Endocrinology, Diabetes, & Metabolism Case Reports*	Bioscientifica	2013	Yes	Yes
*Epilepsy & Behavior Case Reports*	Elsevier	2013	Yes	Yes
*European Journal of Case Reports in Internal Medicine*	European Federation of Internal Medicine	2014	Yes	No
*European Journal of Pediatric Surgery Reports*	Thieme Medical Publishers	2013	Yes	Yes
*European Journal of Surgical Cases*	Bilimsel Tip Yayinevi	2010	Yes	No
*Experimental and Clinical Endocrinology & Diabetes Reports*	Thieme Medical Publishers	2014	Yes	No
*Global Journal of Medical and Clinical Case Reports*	PeerTechz	2014	Yes	No
*Grand Rounds*	e-MED	2001	Yes	No
*Gynecologic Oncology Reports*	Elsevier	2011	Yes	Yes
*HeartRhythm Case Reports*	Elsevier/Heart Rhythm Society	2015	Yes	No
*Human Pathology: Case Reports*	Elsevier	2014	yes	No
*IDCases*	Elsevier	2014	Yes	No
*IJSS Case Reports & Reviews*	IJSS Group of Journals/Society of Malaysian Medical Association’s Medical Students and European Medical Student’s Association	2014	Yes	No
*Indian Journal of Medical Case Reports*	CIBTech	2012	Yes	No
*Interdisciplinary Neurosurgery: Advanced Techniques and Case Management*	Elsevier	2014	Yes	No
*International Journal of Advances in Case Reports*	McMed International	2014	Yes	No
*International Journal of Case Reports and Images*	Edorium Journals	2010	Yes	No
*International Journal of Case Reports in Medicine*	IBIMA Publishing	2012	Yes	No
*International Journal of Case Studies*	unclear	2012	Yes	No
*International Journal of Clinical Case Studies*	Graphy Publications	2014	Yes	No
*International Journal of Clinical Cases and Investigations*	unclear	2010	Yes	No
*International Journal of Medical and Pharmaceutical Case Reports*	ScienceDomain International	2014	Yes	No
*International Journal of Surgery Case Reports*	Elsevier	2010	Yes	Yes
*International Medical Case Reports Journal*	Dove Medical Press	2008	Yes	Yes
*JAAD Case Reports*	Elsevier/American Academy of Dermatology	2015	Yes	No
*Jacobs Journal of Clinical Case Reports*	Jacobs Publishers	2015	Yes	No
*JBJS Case Connector*	STRIATUS Orthopaedic Communications	2011	No	No
*JCRS Online Case Reports*	Elsevier/American Society of Cataract and Refractive Surgery and European Society of Cataract and Refractive Surgeons	2013	Yes	No
*JMM Case Reports*	Microbiology Society	2014	Yes	No
*Joseph Journal of Clinical Studies and Medical Case Reports*	Joseph Publishing Group	2015	Yes	No
*Journal of Anaesthesia & Critical Care Case Reports*	International Academic Research Group	2015	Yes	No
*Journal of Cardiology Cases*	Elsevier/Japanese College of Cardiology	2010	No	No
*Journal of Case Reports*	unclear	2011	Yes	No
*Journal of Case Reports and Clinical Research Studies*	VRJ Publishers	2014	Yes	No
*Journal of Case Reports and Images in Medicine*	Edorium	2015	Yes	No
*Journal of Case Reports and Images in Obstetrics and Gynecology*	Edorium	2015	Yes	No
*Journal of Case Reports and Images in Oncology*	Edorium	2015	Yes	No
*Journal of Case Reports and Images in Pathology*	Edorium	2015	Yes	No
*Journal of Case Reports and Images in Surgery*	Edorium	2015	Yes	No
*Journal of Case Reports and Studies*	Annex Publishers	2013	Yes	No
*Journal of Case Reports in Medicine*	Ashdin Publishing	2012	Yes	No
*Journal of Case Reports in Oncology and Therapy*	EJourPub	2015	Yes	No
*Journal of Case Reports in Practice*	Saman Publishing	2013	Yes	No
*Journal of Clinical & Medical Case Reports*	Avens Publishing Group	2013	Yes	No
*Journal of Clinical and Translational Endocrinology Case Reports*	Elsevier	2015	Yes	No
*Journal of Clinical Case Reports*	OMICS International	2011	Yes	No
*Journal of Clinical Studies & Medical Case Reports*	Herald Scholarly Open Access	2014	Yes	No
*Journal of Dermatological Case Reports*	Specjaliści Dermatolodzy	2007	No	No
*Journal of Investigative Medicine High Impact Case Reports*	SAGE Publications	2013	Yes	No
*Journal of Knee Surgery Reports*	Thieme Medical Publishers	2013	Yes	No
*Journal of Medical Case Reports*	BioMed Central	2007	Yes	Yes
*Journal of Medical Cases*	Elmer Press	2010	Yes	No
*Journal of Neurological Surgery Reports*	Thieme Medical Publishers	2012	Yes	Yes
*Journal of Orthopaedic Case Reports*	Indian Orthopaedic Research Group	2011	Yes	No
*Journal of Pediatric Surgery Case Reports*	Elsevier	2013	Yes	No
*Journal of Radiology Case Reports*	EduRad Publishing	2008	Yes	Yes
*Journal of Surgical Case Reports*	Oxford University Press	2010	Yes	Yes
*Journal of Surgical Technique and Case Report*	Wolters Kluwer Health	2014	Yes	Yes
*Journal of Vascular Surgery Cases*	Elsevier/Society for Vascular Surgery	2015	Yes	No
*JPRAS Open*	Elsevier/British Association of Plastic Reconstructive and Aesthetic Surgeons	2015	Yes	No
*JSM Clinical Case Reports*	JSciMed Central	2013	Yes	No
*Medical Case Studies*	Academic Journals	2010	Yes	No
*Medical Mycology Case Reports*	Elsevier/International Society for Human and Animal Mycology	2012	Yes	Yes
*MOJ Clinical & Medical Case Reports*	MedCrave	2015	Yes	No
*Neurocase*	Taylor & Francis	1995	Optional	Yes
*NMC Case Report Journal*	Japan Neurosurgical Society	2014	Yes	No
*OA Case Reports*	OA Publishing London	2012	yes	No
*Oncology & Cancer Case Reports*	OMICS International	2015	Yes	No
*Open Journal of Clinical and Medical Case Reports*	unclear	2015	Yes	No
*Oral and Maxillofacial Surgery Cases*	Elsevier	2015	yes	No
*Oxford Medical Case Reports*	Oxford University Press	2014	Yes	Yes
*Pathology Case Reviews*	Wolters Kluwer Health	1996	No	No
*Pediatric Urology Case Reports*	Hayrettin Ozturk	2014	Yes	No
*Radiology Case Reports*	Elsevier/University of Washington	2006	Yes	No
*Respiratory Medicine Case Reports*	Elsevier	2008	Yes	Yes
*Respirology Case Reports*	Wiley/Asian Pacific Society of Respirology	2013	Yes	Yes
*Retinal Cases and Brief Reports*	Wolters Kluwer Health	2007	Optional	Yes
*SAGE Open Medical Case Reports*	SAGE Publications	2013	Yes	Yes
*Scholarena Journal of Case Reports*	Scholarena	2014	Yes	No
*Scholars Journal of Medical Case Reports*	SAS Publishers	2013	Yes	No
*Southeast Asian Journal of Case Report and Review*	Sageya Publishing	2012	Yes	No
*Surgical Case Reports*	Springer/Japan Surgical Society	2015	Yes	No
*The Thoracic and Cardiovascular Surgeon Reports*	Thieme Medical Publishers	2014	Yes	Yes
*Translational Medicine Case Reports*	Elsevier/European Society for Translational Medicine	2015	Yes	No
*Trauma Case Reports*	Elsevier	2015	Yes	No
*Urology Case Reports*	Elsevier	2013	yes	No
*World Journal of Clinical Cases*	Baishideng Publishing Group	2013	Yes	Yes
*World Journal of Medical and Surgical Case Reports*	Narain Publishers	2012	Yes	No

1. The error: ‘The journal title *BMJ Case Reports* has no value in the PubMed Indexed column‘ Should instead read: ‘*BMJ Case Reports* in the PubMed Indexed column should have indicated “Yes”because it is PubMed indexed‘

2. The error: ‘The journal title *BMJ Case Reports* has value “No” in the Open access column‘ Should instead read: *‘BMJ Case Reports* in the Open access column should have indicated “Optional” because it does have an option for open access for an extra fee‘

This has now been included in this erratum.
